# 208. Comparison of Bloodstream Infections in Hospitalized Patients Before and During the COVID-19 Surge in a Community Hospital in the South Bronx: An Observational Study

**DOI:** 10.1093/ofid/ofab466.410

**Published:** 2021-12-04

**Authors:** Afsheen Afzal, Edgar Gomez, Victor Perez Guttierrez, Aye Myat Mon, Carolina Moreira Sarmiento, Amna Khalid, svetlana Polishchuk, Mohannad Al-Khateeb Al-Khateeb, Boyana Yankulova, Mubarak Yusuf, Yinelka Silverio De Castro, Anjana Pillai, Usha Venugopal, Addi Feinstein, Alexander LaFortune, Daniel Sittler, Karen Hennessey, Vidya Menon

**Affiliations:** Lincoln Medical Center, New York, New York

## Abstract

**Background:**

There is a paucity of data of bloodstream infections (BSI) before and during the COVID-19 pandemic. The aim of our study was to compare the incidence and characteristics of blood stream infections (BSI) in hospitalized patients before and during the surge of COVID-19 pandemic in a community hospital in South Bronx.

**Methods:**

This is a retrospective observational comparative study of adult hospitalized patients with BSI admitted before (Jan 1-Feb 28, 2020) and during COVID-19 surge (Mar 1- May 1,2020). The incidence of BSI, patient demographics, clinical and microbiological characteristics of infections including treatment and outcomes were compared.

**Results:**

Of the 155 patients with BSI, 64 were before COVID and 91 were during the COVID surge (Table 1). Incidence of BSI was 5.84 before COVID and 6.57 during surge (p = 0.004). Majority of patients during COVID period had ARDS (39.6%), required mechanical ventilation (57%), inotropic support (46.2%), therapeutic anticoagulation (24.2%), proning (22%), rectal tube (28.6%), Tocilizumab (9.9%), and steroids (30.8%) in comparison to pre-COVID (Table 2). Days of antibiotic therapy prior to BSI was 5 days before COVID and 7 during COVID. Mortality was higher among patients with BSI admitted during COVID surge (41.8% vs. 14.1% p < 0.0001). Of 185 BSI events, 71 were Pre-COVID and 114 during surge. Primary BSI were predominant (72%) before COVID contrary to secondary BSI (46%) (CLABSI) during COVID. Time from admission to positive culture was 2.5 days during COVID compared to 0.9 pre-COVID. Majority of BSI during COVID period were monomicrobial (93%) and hospital acquired (50%) (p=0.001). *Enterococcus* (20.2%), *E.coli* (13.2%), and *MSSA* (12.3%) were predominant microbes causing BSI during COVID vs. *MRSA* (15.5%), *Streptococci* (15.5%), and *S. pneumoniae* (14.1%) before COVID (Figure 1). In multivariate logistic regression, *Enterococcal* coinfection was associated with COVID positivity (OR 2.685, p = 0.038), mechanical ventilation (OR 8.739, p = 0.002), and presence of COPD/Asthma (OR 2.823, p = 0.035).

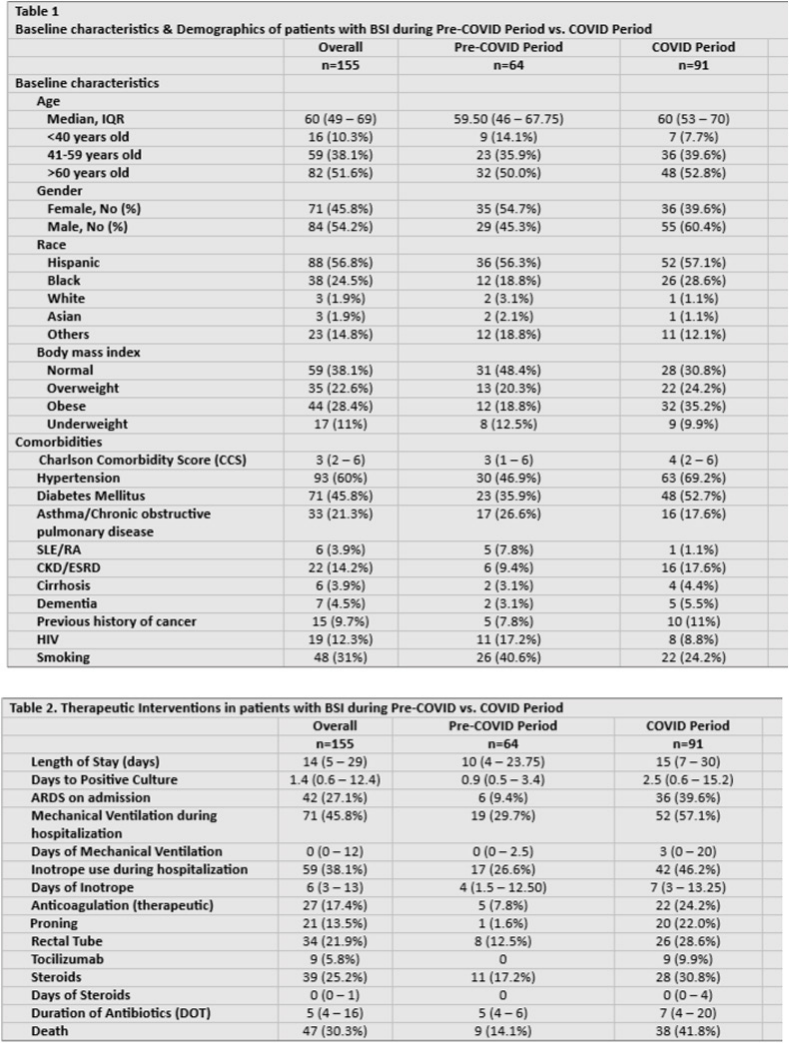

Comparison of Microorganisms Isolated in the BSI

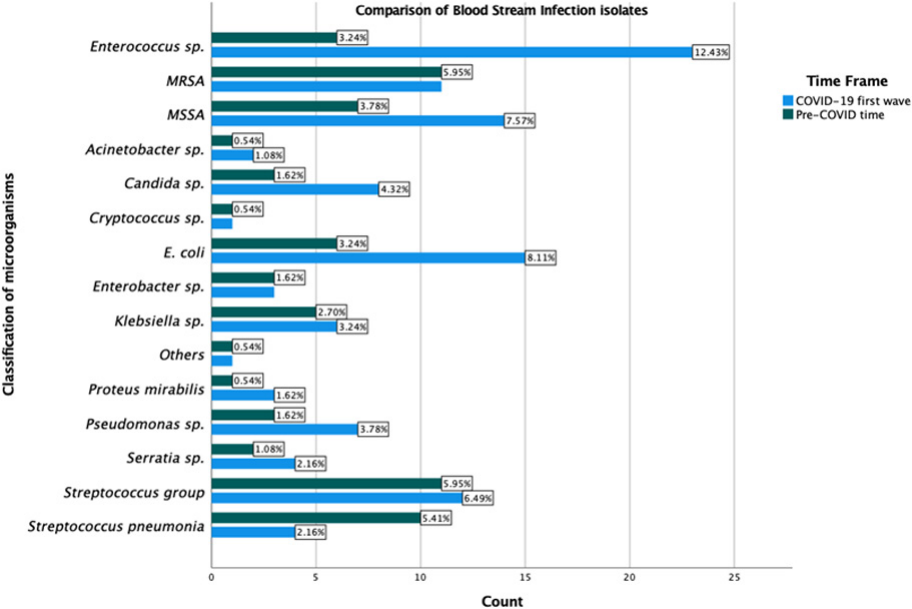

X-axis represents the total number of BSI events whereas the number at the end of each bar represents the percentage

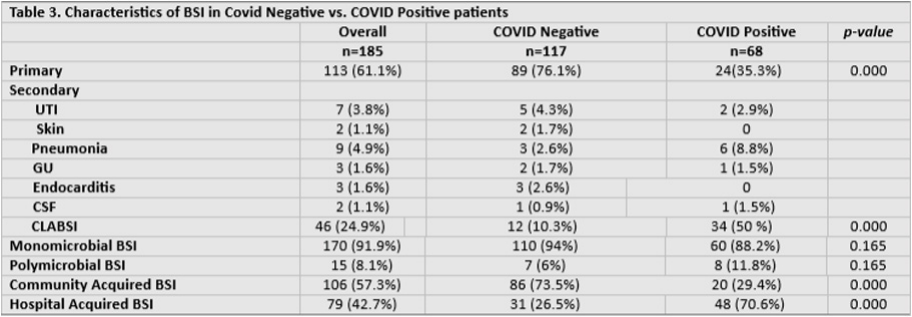

**Conclusion:**

Higher incidence of secondary BSI (CLABSI) due to *Enterococcus spp*. was observed during the surge of COVID-19 infection in the South Bronx. Breakdown of infection control measures during the COVID-19 pandemic could have been contributory.

**Disclosures:**

**All Authors**: No reported disclosures

